# Radiation Safety and Future Innovative Diagnostic Modalities

**DOI:** 10.1155/2008/827106

**Published:** 2008-06-04

**Authors:** Christian Radmayr

**Affiliations:** Department of Pediatric Urology, Medical University Innsbruck, 6020 Innsbruck, Austria

## Abstract

One must demand an accurate, safe, radiation-free, and noninvasive method for reflux examination as the ideal possibility for reflux screening. Of course the available different imaging modalities are far from this ideal situation, but minimal radiation exposure is indeed a permanent objective. Additionally since all of these studies might be quite stressful to the child and the family, a specially designed and equipped environment is obligatory for the comfort of all involved. An absolute ideal modality in the diagnosis of VUR would be the definition of a certain marker in serum or urine that could identify children with VUR without the need for any interventional screening modality. Therefore more and more efforts have to be made in the future to investigate different markers for this purpose. Since reflux is one of the most frequent congenital conditions pediatric urologist have to deal with potential risks that might lead to renal insufficiency, noninvasive and radiation-free modalities should become the methods of choice, hopefully in the near future.

## 1. INTRODUCTION

Different imaging modalities for evaluating vesicoureteral reflux (VUR) are nowadays available and more and more attention is paid to concerns about radiation safety, especially when multiple imaging studies are necessary with repeat exposition to ionizing radiation during a conservative follow up. Minimal radiation exposure is now a permanent objective.

Experience and logistic availability are often responsible for choosing the one or the other imaging study. Moreover, patient age, gender, race, and parental preference and anxiety about invasiveness and radiation exposure play an additional role as well.

In the past years, different innovative techniques like low-dose fluoroscopy have definitively decreased radiation dose to the patient. On the other hand, diagnostic quality images are mandatory for appropriate diagnosis and treatment. Ultrasonography, nuclear medicine, and magnetic resonance are preferred to intravenous urography and computed tomography whenever possible. In general, voiding cystourethrography has frequent indications in pediatric urology and efforts are made to replace it by radionuclide cystograms or sonocystograms in order to reduce the exposure to ionizing radiation.

Furthermore, there is also no consensus about the timing of evaluation for possible reflux as well as in the conservative follow up or after intervention.

## 2. VOIDING CYSTOURETHROGRAPY, RADIONUCLIDE CYSTOGRAPHY, SONOCYSTOGRAPHY

All these three modalities can be used to demonstrate the presence or absence of VUR. Voiding cystourethrograpy (VCUG) as well as direct radionuclide cystography (RNC) are invasive diagnostic tools with the need for patient preparation and catheterization and the exposition of the patient to ionizing radiation although VCUG is a fluoroscopic examination and RNC a nuclear medicine study, respectively. Additionally, an indirect RNC with the intravenous administration of technetium-99m-labeled diehtylenetriamine pentaacetic acid is a possible tool with the assumption of a possible VUR when radioisotope counts increase in the renal areas after voiding. But the false negative rates may vary between 22 to 51% [1].

Even though being invasive as well since catheterization is mandatory, the big advantage of performing a sonocystography is the prevention of any ionizing radiation to the patient at all.

For a conventional VCUG, a water soluble contrast medium is instilled into the bladder after preparation and transurethral sterile catheterization. An option is the suprapubic administration of the contrast, but still invasive. Different fluoroscopic images are taken to demonstrate the presence or absence of vesicoureteral reflux.

The same procedure (preparation and catheterization) is necessary for performing an RNC. Usually technetium 99m pertechnetate is the radiopharmacon of choice to be instilled into the bladder. The radioactive emissions are continuously recorded with a gamma camera.

When comparing these two wide spread diagnostic modalities, the big advantages of a classical VCUG are the provision of high-resolution images with a clear evaluation of the bladder wall, the urethra especially in males [2], any sign of intrarenal reflux as well as a clear grading possibility. It is also supplementary reliable for detecting duplication, ureteral ectopia with or without ureteroceles, and posterior urethral valves. That is why especially in boys and also for the initial investigation in girls a classical VCUG is still the preferred method of choice by many investigators. On the other hand, the expense is a much higher dose of ionizing radiation to the patient.

Although recent improvements introducing low-dose fluoroscopy techniques and pulse fluoroscopy with the add of digital enhancing modalities have decreased the radiation dose to the patients dramatically [3–5], still a VCUG exposes the patient to almost 100 times the radiation of an RNC. A special concern is the quite high gonadal radiation dose particularly with multiple studies of fluoroscopic monitoring [6]. Of course gonadal shielding in males and careful imaging coning help to decrease the patient's radiation exposure. Moreover, with the use of a low-dose fluoroscopic system in conjunction with a computer-based video frame grabber, the ovarian radiation dose may become comparable to RNC [3]. A VCUG performed with an optimized pulsed fluoroscope can achieve “as low as reasonably achievable” (ALARA) levels and of course maintain diagnostic image quality. With such a setting radiation dosage can be reduced to 10% that of continuous fluoroscopy thus resulting in dosages at about 10 times that of RNC. Therefore, pulsed fluoroscopy is currently the recommended standard [7, 8].

On the other hand, a direct RNC allows continuous monitoring for VUR throughout the whole examination time without any additional radiation introduced. Therefore, some authors prefer RNC to be more sensitive in the diagnosis of VUR [9] although precise grading is impossible. But this makes it probably an ideal methodology for the conservative follow up and after any antireflux intervention.

The main advantage of RNC over fluoroscopic VCUG is definitively decreased radiation exposure of the patient. The average effective radiation dose of a VCUG using low-dose fluoroscopy is around 3 mrem, compared to 0.5 mrem for an RNC. Of course the average effective dose of the VCUG is variable and depends on the patient size, operator, and machinery [8]. The sensitivity of RNC for detecting reflux is equal to or even greater than that of VCUG; however, the spatial resolution and anatomic detail seen on an RNC are ultimately inferior to those seen on a VCUG [10].

Sonocystography may be used as a very sensitive tool in the detection of a possible VUR especially since the intervention of various ultrasound echo enhancing agents [11]. First, attempts with this technology have been made back in 1976. The capability of echo-enhanced refluxsonography extends further in that the method may enable complete elimination of any radiation exposure. This may justify the longer examination time compared with that of VCUG. Using an X-ray contrast agent, a certain concentration at a given time is necessary to be able to see the contrast, whereas even single microbubbles can be visualized with the ultrasound method. This together with the duration of the ultrasound examination as well might be responsible for the detection of some low grade refluxes that might be missed using VCUG and RNC. Moreover, this method allows for cyclic fillings without any additional radiation as well. On the other hand, similar to RNC, the lack of diagnostic visualization of anatomic details and particularly the urethra represent a disadvantage of the ultrasound methodology. Additionally, the interobserver variability might be quite high and a specially trained examiner is obligatory. In summary, of the available literature on that issue, the comparative aggregated data between sonocystography and VCUG indicate that reflux exclusion and diagnosis between the two methods is highly concordant and that the discordant findings are primarily due to more reflux episodes being detected solely by sonocystography and that these reflux episodes are of higher grade and consequently may be clinically more relevant than the predominantely low grade reflux found only on VCUG and that finally the high negative predictive value of sonocystography may have practical consequences as it demonstrates that sonocystography may be suitable for screening purposes [12, 13].

## 3. CONCLUSION AND FUTURE MODALITIES

One must demand an accurate, safe, radiation-free, and noninvasive method for reflux examination as the ideal possibility for reflux screening. Additionally, since all of these studies might be quite stressful to the child and the family a specially designed and equipped environment is obligatory for the comfort of all involved. Preparation and education of the families help to reduce discomfort. If needed, sedation with the use of midazolam can be beneficial without any negative influence on the outcome of the examination [14].

Contrast enhanced ultrasound allows an accurate and safe diagnosis and is in addition to VCUG and RNC radiation free as well; but unfortunately, still an invasive procedure with the insertion of a catheter. A future prospective might be an exogenous bubble generation to fulfil one of the most important criteria in reflux diagnosis: being noninvasive. Efforts are already being made to achieve this goal. Till then nuclear medicine studies and contrast studies will remain essential for the evaluation of VUR.

An absolute ideal modality in the diagnosis of VUR would be the definition of a certain marker in serum or urine that could identify children with VUR. Basic research is going on to investigate different markers that have been found to be elevated in children with VUR [15]. Measured levels of microproteinuria, urine retinol-binding protein, urinary prostaglandine E_2_, urinary *β*_2_-microglobulin, urinary interleukin levels, and serum endothelium leukocyte adhesion molecule have been shown to be elevated in patients with VUR compared to controls. So far, none of these methods can localize which kidney is affected by reflux nor can they assess the grade but they probably offer the potential advantage of rapidly screening for VUR.

Another marker, *β*-hexosaminidase, has been shown to be higher in patients with VUR and renal scarring [16]. Tamm-Horsefall protein (THP) is another high-molecular-weight glycoprotein that is exclusively present in the kidney and not secreted elsewhere. In children with intrarenal reflux, it is also detectable in blood vessels and lymph nodes. It is believed to accumulate from leakage of adjacent ruptured tubules [17]. Interestingly, in a study on children with surgically corrected VUR but no improvement on renal function postoperatively, THP levels remained elevated before and after surgery [18]. Still a lot of research has to be undertaken to minimize or hopefully abandon the burden of one of the widest used imaging modalities in pediatric urology.
